# Efficacy and Safety of Treatments for Primary Palmar Hyperhidrosis: A Systematic Review Assessing Patient-Centric Outcomes

**DOI:** 10.1155/drp/8867838

**Published:** 2025-08-20

**Authors:** Foteini Moniati, Marianna Vassiliou, Christos Costa, Constantina Chatzimatthaiou, Marios Chatzimatthaiou

**Affiliations:** ^1^Faculty of Medicine, University Hospitals Birmingham NHS Foundation Trust, Birmingham, UK; ^2^Faculty of Medicine, Barts and the London School of Medicine and Dentistry, Queen Mary University of London, London, UK; ^3^Faculty of Medicine, Imperial College London, London, UK; ^4^Faculty of Medicine, Medway NHS Foundation Trust, Gillingham, UK

**Keywords:** botulinum toxin, compensatory hyperhidrosis, endoscopic thoracic sympathectomy, iontophoresis, oxybutynin, photodynamic therapy, primary palmar hyperhidrosis, systematic review

## Abstract

**Background:** Primary palmar hyperhidrosis (PH) is a chronic condition characterized by excessive sweating in the palms, significantly affecting the quality of life (QOL) of affected individuals. Despite the availability of various treatment modalities, the long-term efficacy and safety of these interventions remain unclear, warranting a comprehensive evaluation. This systematic review aims to assess the efficacy, safety and patient-reported outcomes of treatments for PH.

**Methods:** A systematic search was conducted in PubMed, Embase and the Cochrane Library from their inception until March 2024, adhering to PRISMA guidelines. Inclusion criteria focused on prospective and retrospective studies examining PH treatments published in English. Data from eligible studies were extracted, analysed qualitatively and reported based on outcomes, including efficacy, QOL improvements and adverse effects.

**Results:** Fourteen studies, including 1733 patients aged 4–77 years, were included in the final review. The treatments assessed included oral and topical oxybutynin, iontophoresis, botulinum toxin A injections, photodynamic therapy (PDT) and endoscopic thoracic sympathectomy (ETS). Oral oxybutynin demonstrated symptomatic relief in 60%–97% of the patients although anticholinergic side effects were frequently reported. ETS, while providing the highest rates of complete sweat cessation, was associated with compensatory hyperhidrosis. Noninvasive treatments like iontophoresis showed moderate efficacy with minimal side effects but required ongoing sessions for maintenance.

**Conclusion:** This review highlights the efficacy of several therapeutic approaches for PH though most treatments are hindered by significant adverse effects or practical limitations. Future research should prioritize long-term studies and standardized outcome measures to guide clinical decision-making more effectively.

## 1. Introduction

Hyperhidrosis is a disorder marked by excessive sweating that surpasses typical physiological requirements, impacting approximately 1%–3% of the population in the UK, particularly individuals aged 18–54 years [1, 2]. Despite its relatively low prevalence, palmar hyperhidrosis (PH) significantly impairs daily functioning and psychosocial wellbeing, underscoring the importance of evaluating treatment outcomes through a patient-centred lens. This condition manifests in two primary forms: idiopathic primary hyperhidrosis and secondary hyperhidrosis, each with unique triggers and implications [3–5]. Secondary hyperhidrosis frequently arises due to underlying medical conditions, including febrile illnesses, neurological disorders such as Parkinson's disease, metabolic abnormalities like hyperthyroidism or the use of certain medications, such as antidepressants and antipsychotics. In contrast, primary hyperhidrosis is believed to result from overstimulation of the sympathetic autonomic nervous system although its exact aetiology remains unclear [6].

Among its various subtypes, primary PH affects approximately 0.6%–1% of the population and is characterized primarily by excessive sweating of the hands and palms, which can significantly hinder daily activities and diminish the quality of life (QOL) [7]. PH typically emerges in late childhood or early adolescence and often continues into adulthood, frequently coexisting with other forms of hyperhidrosis, including plantar and axillary hyperhidrosis. Although histopathological studies have documented features indicative of sweat gland hyperactivity, the precise mechanisms underlying PH remain inadequately understood [8, 9].

Management of PH often involves identifying and addressing exacerbating factors prior to initiating treatment. Beyond the physical manifestations, PH profoundly affects psychological wellbeing, leading to social anxiety and embarrassment in situations such as handshakes [9]. In addition, it may contribute to dermatological complications, including dermatitis, skin maceration and secondary infections [7].

The current treatment algorithm for PH typically begins with topical aluminium chloride. Iontophoresis is recommended as a second-line therapy for those with inadequate response. Botulinum toxin A injections are used as a third-line option due to their high efficacy though the effects are temporary and require repeat treatments. If symptoms persist, oral anticholinergics such as oxybutynin may be considered, especially in patients with multifocal disease or severe symptoms. Endoscopic thoracic sympathectomy (ETS) is reserved for refractory cases due to the high risk of compensatory hyperhidrosis. However, existing guidelines, including the S1 consensus, do not yet incorporate newer treatments such as topical oxybutynin gel, combination therapies or photodynamic therapy (PDT) [10]. These emerging options have shown encouraging results in recent studies. This gap highlights the need to update current guidelines to reflect new evidence, better address multifocal presentations and support more tailored, patient-centred treatment strategies.

Given these complexities, a holistic treatment strategy is essential for mitigating the adverse effects of PH on patients' social, emotional and physical health. By addressing both the physiological and psychological dimensions of hyperhidrosis, healthcare professionals can enhance support for individuals living with this challenging condition [11, 12]. Within this context, the objective of this article is to thoroughly examine all published studies that look into treatment modalities for PH. The efficacy and safety of these modalities will be thoroughly reviewed through a systematic review and evaluation, providing a detailed overview of their outcomes based on the existing scientific literature.

## 2. Methods

### 2.1. Literature Search Strategy

The Preferred Reporting Items for Systematic Reviews and Meta-Analyses (PRISMA) checklist and flow diagram were followed. The systematic search of the online databases PubMed, Embase and Cochrane Library was conducted from inception until 1^st^ March 2024 to identify all relevant studies. The search terms employed were as follows: (“Hyperidrosis” OR “Palmar Hyperidrosis” OR “PH”) AND (“Oxybutynin” OR “Botulinum” OR “Botulinum Toxin” OR “Botox” OR “Antimuscarinic” OR “Anticholinergic” OR “Iontophoresis” OR “Photodynamic Therapy” OR “PDT” OR “Endoscopic Thoracic Sympathectomy” OR “Thoracic Sympathectomy” OR “ETS”)).

### 2.2. Inclusion and Exclusion Criteria

Our study employed specific inclusion criteria, with a primary focus on research pertaining to the treatment of PH. We narrowed our search to prospective and retrospective studies published in English and available as complete articles in peer-reviewed journals. Exclusion criteria comprised literature reviews, conference papers, animal trials, registered trials and studies lacking full-text accessibility.

### 2.3. Data Extraction and Quality Assessment

The process of study selection was carried out independently by two of the authors (F.M and C.C) at the outset. A consensus on the research question was reached, and MeSH terms were employed for the screening process. Initially, the authors screened the titles and pertinent abstracts, and when further evaluation was deemed necessary, the entire paper was comprehensively reviewed. In cases where disagreements arose concerning the inclusion of particular studies, a third independent author (C.H) was consulted. This author implemented a voting system to determine whether the study in question should be incorporated or excluded from the analysis [13].

Two independent reviewers diligently collected and transcribed data from the eligible studies into an electronic screening form. The data extracted from each article encompassed various aspects, including authors' names, study title, publication year, study type, sample size, intervention, outcome measures, evaluation period and outcomes including adverse effects [13]. A qualitative synthesis was selected over a meta-analysis due to significant heterogeneity among the available studies in terms of study design, patient populations, interventions and outcome measures, which precluded meaningful quantitative pooling of data.

Methodological quality and risk of bias involved the application of the Cochrane Collaboration's tool [14]. Each checklist item was assigned 0 points for “No,” 1 point for “Unclear,” and 2 points for “Yes.” Subsequently, total scores were tabulated and expressed as a percentage of the overall score. Studies were categorized as exhibiting low, moderate or high risk of bias if the percentage of the total score equated to ≥ 80%, 60%–80% or ≤ 60%, respectively.  Table 2.0 illustrating the methodological quality of the studies can be found in the Supporting section.

## 3. Results

### 3.1. Description of the Studies

**Figure 1:**
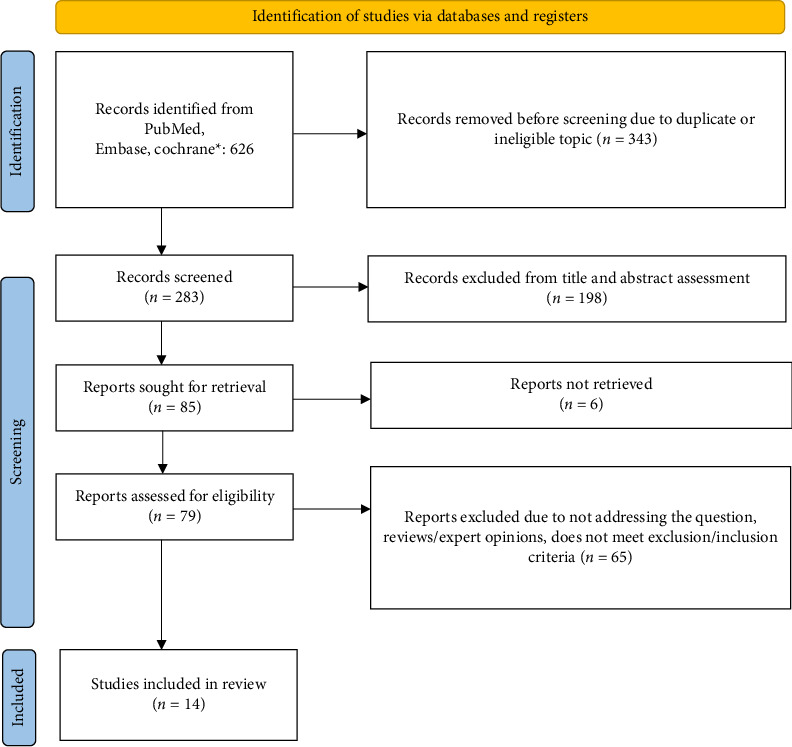
PRISMA flow diagram of the selected studies.

There were 626 studies collated in the initial database search. After the removal of duplicates, ineligible by topic records or those removed for other reasons, the number was subsequently reduced to 283. Following abstract screening, 79 reports were assessed for eligibility, leaving 14 studies, published between 1997 and 2023, included in the final review. The total number of all participants included in the fourteen studies in this review was 1733, with ages ranging from 4 to 77 years. The PRISMA flowchart with reasons for exclusion is illustrated in Figure 1.

### 3.2. Intervention

The review highlights the use of five different therapies for PH summarized in Table 1. These therapies include oxybutynin as an anticholinergic treatment, iontophoresis with glycopyrronium bromide, PDT, intradermal botulinum toxin A palmar injections and ETS. Out of the 14 studies, 5 investigated the use oral oxybutynin, while 2 studies investigated the use of topical oxybutynin. One study carried out by Campanati et al., specifically, looked into the use of oral oxybutynin for relapse management compared with first-line treatment. The use of iontophoresis was investigated in two studies, the use of PDT in only one study, while the use of botulinum toxin A injections was investigated in two studies. One of these studies was the aforementioned investigation by Campanati et al. that used botulinum toxin A injections as the first line in the arm that was administered oxybutynin a relapse. Finally, ETS was tested in the rest of the studies (*n* = 3). One of these studies video-assisted bilateral ETS of the second and third ganglia (T2-T3), the other study carried out ETS by ablation of the second, third and fourth ganglia (T2–T4) by electrocauterization and the final study performed bilateral and sequential ETS using an electrocoagulation hook at level T4 and extended 2 cm laterally at levels T3 and T4 to include the Kuntz's nerve. Alternative therapies such as glycopyrrolate and aluminium chloride were excluded due to insufficient robust evidence and limited clinical utilization within the targeted population.

### 3.3. Outcome Assessment

The methods of outcome assessment varied between the studies. However, the most common assessments seen in all 14 studies were subjective patient recording questionnaires or scales to assess outcome measures, including quality of life questionnaires (QOLQs), the Hyperhidrosis Disease Severity Scale (HDSS), the Visual Analogue Scale (VAS), the Dermatology Life Quality Index (DLQI) and other surveys of patient satisfaction, sweat reduction and side effects. Clinical follow-up questionnaires were also carried out in two studies in addition to the patient questionnaires. Blinded visual grading of photographs taken before and after treatment was another subjective method of recording treatment outcomes used in one of the studies.

Objective assessment of patient outcomes and specifically sweat reduction was seen in only five of the fourteen studies. Two of these studies utilized the sweating intensity visual scale with Minor's test, also known as the Minor's iodine starch test and two other studies utilized colorimetric and/or gravimetric measurements to assess treatment success. The remaining study used digital ninhydrin sheets as an objective measurement of sweat reduction. Both objective and subjective tests were carried out at the baseline and at the end of treatment in most studies. Time to follow-up appointments was recorded in 9 of the studies.

### 3.4. Adverse Effects

All studies reported improvements following treatment administration and two out of the fourteen studies reported complete or near-complete resolution of the symptoms. Adverse effects were reported in all studies with most symptoms being classified as mild and self-limiting. The most common adverse effect reported in 5 studies was dry mouth, followed by compensatory hyperhidrosis reported in 3 studies. Symptoms involving the hands included transient discolouration and mild localized erythema, as well as mild weakness and grip. More general symptoms like anticholinergic side effects, urinary retention, bodily pains, transient headaches and pruritus were also reported. The study with the most serious side effects was the one carried out by Campanati et al., where eleven patients withdrew following symptoms like xerophthalmia, vaginal dryness, dizziness and constipation.

## 4. Discussion

This systematic review aimed to evaluate the efficacy and safety of various treatments for primary PH, a condition that severely impairs patients' QOL. Fourteen studies published between 1997 and 2023, encompassing 1733 patients, were analysed, covering both noninvasive and surgical treatment options. The findings reveal significant therapeutic benefits but also underscore notable limitations, particularly in terms of adverse effects, variability in outcomes and research design inconsistencies. Overall, this review provides crucial insights into the clinical management of PH and also highlights areas for improvement.

The therapies analysed varied widely in terms of efficacy. Oral oxybutynin, evaluated in five studies, consistently showed improvements in patient-reported outcomes, with 60%–97% of the patients experiencing symptomatic relief. However, the frequent occurrence of anticholinergic side effects such as dry mouth and urinary retention presents a challenge for long-term use [24]. Despite its efficacy, the side effects often led to patient discontinuation, particularly at higher doses [22]. Concurrently, whilst effective, oral oxybutynin is associated with central nervous system side effects, highlighting the need for peripherally acting agents that do not cross the blood–brain barrier. The study by Campanati et al. (2020) provided valuable insights by comparing relapse management using a combination of botulinum toxin A and oral oxybutynin, which showed better control over relapsing symptoms compared with monotherapy. This suggests that combination therapies may enhance long-term outcomes although more robust data are required to validate these findings.

The recent observational study by Markantoni et al. (2023) provides valuable insights into the clinical characteristics and management of multifocal hyperhidrosis (MFH), a phenotype of primary focal hyperhidrosis that often includes palmar involvement. Despite its significant impact on QOL, MFH remains underrepresented in the literature, particularly compared with more localized forms such as isolated palmar or axillary hyperhidrosis. This study, which included 102 patients with MFH, highlights the high prevalence of palmar/plantar and axillary involvement, the early onset of symptoms and the frequent exacerbation by environmental and emotional triggers. Notably, the authors report high treatment persistence with oral oxybutynin and advocate for its early use as a systemic therapy in MFH, an approach not yet emphasized in current guidelines. These findings contribute novel epidemiological and therapeutic perspectives on MFH, reinforcing the need to recognize this subtype as a distinct and burdensome clinical entity that warrants targeted research and tailored management strategies.

Botulinum toxin A injections have demonstrated high efficacy in reducing palmar sweating, with consistent improvements in both HDSS and QOL scores across studies [19, 28]. Despite these benefits, several limitations affect its long-term practicality. The effects are temporary, requiring repeated administrations, and the treatment is associated with high costs. In addition, pain during injection is a significant patient concern, often influencing treatment satisfaction and willingness to continue therapy. As reported by Kanni et al., this discomfort can be substantial enough to reduce adherence, particularly in patients seeking long-term relief [29]. While botulinum toxin A remains an effective noninvasive option, its limitations underscore the need for more durable, accessible and better-tolerated therapies, particularly for those with multifocal disease or requiring sustained symptom control.

Endoscopic thoracic sympathectomy, the most invasive intervention in this review, showed the highest rates of complete resolution of palmar sweating [17, 23]. However, its efficacy comes at the cost of compensatory hyperhidrosis, which affected up to 94.6% of the patients in some studies [17]. This adverse effect, characterized by excessive sweating in areas such as the back or legs, can be as debilitating as the original condition and often leads to postsurgical dissatisfaction. Given the high incidence of compensatory sweating and the lack of effective solutions to mitigate it, ETS should be reserved for highly selected cases after a thorough discussion of risks. These findings align with previous research that suggests ETS is highly effective but controversial due to its long-term side effects [22, 27].

Iontophoresis, evaluated in two studies, emerged as a safer but less potent treatment option. Both studies showed improvement in sweat reduction and QOL, with minimal side effects such as mild erythema and localized irritation [20, 25]. While its noninvasive nature and tolerability make it an appealing option for patients with moderate symptoms, the need for frequent sessions and lower efficacy compared with other therapies may limit its use in severe cases. Similarly, PDT, though associated with minimal side effects, lacks robust evidence of long-term efficacy, with only one study addressing its use for PH [18]. This highlights the need for further research to validate PDT as a viable treatment alternative.

This systematic review presents several notable limitations that warrant consideration. A primary limitation lies in the considerable heterogeneity among the included studies, both in terms of study design and the measures used to assess outcomes. Many studies relied predominantly on subjective self-reported instruments, such as the HDSS and QOLQs, which may introduce bias and impede the comparability of results across interventions. The lack of consistency in outcome measures limits comparability between interventions and highlights the need for standardized, validated tools to assess both symptom burden and treatment benefit in future research. Objective assessments, such as gravimetry and Minor's iodine starch tests, were less frequently employed, thereby limiting the ability to rigorously quantify treatment efficacy. Furthermore, the short duration of follow-up in many studies constrains the assessment of the long-term durability of treatment outcomes, particularly for interventions like botulinum toxin A and iontophoresis, which require ongoing management. The exclusion of non-English language studies and nonpeer-reviewed literature also poses a potential source of publication bias, as relevant data may have been overlooked. Finally, the underrepresentation of key demographic groups, including older adults and individuals with comorbid conditions, limits the external validity of the findings, reducing their applicability to diverse clinical populations. These limitations underscore the need for more standardized, long-term and inclusive research in this field.

## 5. Conclusion

In essence, while this systematic review highlights several effective treatments for PH, it also exposes critical limitations in both the therapies and the evidence supporting them. Oral oxybutynin and ETS remain among the most effective therapies but are hindered by notable adverse effects, including anticholinergic toxicity and compensatory hyperhidrosis, respectively. Less invasive interventions such as iontophoresis and botulinum toxin A injections present promising alternatives but are limited by either reduced efficacy or the need for repeated administration and associated patient discomfort.

As authors, we believe the current therapeutic paradigm for PH remains insufficiently responsive to the needs of diverse patient populations, especially those with multifocal disease. Our findings underscore the necessity for more durable, patient-centred solutions that prioritize both functional improvement and QOL. There is a clear gap in current clinical guidelines, which do not yet integrate emerging therapies, such as topical oxybutynin gel, combination regimens and PDT, that have demonstrated encouraging preliminary outcomes.

Future research should focus on standardizing outcome measures, conducting long-term follow-up studies and exploring combination therapies to improve the sustainability of treatment effects. In addition, more research is needed to address the high incidence of adverse effects and to develop novel therapies that strike a better balance between efficacy and safety. Until these gaps are addressed, clinicians must navigate these treatment options with caution, always keeping the patient's QOL at the forefront of decision-making.

## Figures and Tables

**Table 1:** Summary of the selected studies.

Study (year)	Study type	Sample size	Age (years)	Intervention	Outcome measures	Reported results	Adverse effects	Extra notes
Markantoni et al. 2023 [15]	Observational retrospective study	102	Mean age was 42.48 ± 25.66 years	Oral oxybutynin ± botulinum toxin A injection + topical anticholinergics	Patient HDSS and treatment duration and tolerability	Overall increased sweat reduction with oral oxybutynin, with a survivability over 2 years for 76.2% of the patients with most common sites: palms/soles (80.4%) and axillae (62.7%)	Not systematically reported, but systemic anticholinergic therapy requires monitoring for cognitive disorders	Patients included had multifocal hyperhidrosis (MFH)

Saki et al. 2023 [16]	Double-blind randomised controlled trial	30	Age ranged from 18 to 49 years with a mean age of 28.86 years	1% oxybutynin topical gel or 1% oxybutynin topical nanoemulgel every 12 h for one month	Patient HDSS, VAS and DLQI	Gel and nanoemulgel groups were similar in HDSS, VAS, and DLQI scores. Both improved patient QOL with no significant statistical difference	Anticholinergic side effects [6] Dry mouth [3] Urinary retention [1]	Patients were assessed before treatment and at the end of study

Zhang et al. 2022 [17]	Long-term retrospective	367	Mean age was 25.1 ± 4.5 years	Endoscopic thoracic sympathectomy (ETS)	QOLQ before and after treatment. Evaluation of hyperhidrosis in each area before/after surgery Satisfaction with surgical results Compensatory hyperhidrosis postoperative	Palmar sweating disappeared in 60.22% of the patients and alleviated in 36.1% of the patients. 90.7% of the patients reported improved QOL after surgery. 24.5% were very satisfied, 44.4% satisfied, 4.9% were not satisfied after procedure. Compensatory hyperhidrosis present in 94.6% cases after ETS	Compensatory sweating Bodily pain	Median follow-up of 14 months

Shabaik et al. 2021 [18]	Single-centre clinical study	20	Age ranged from 15 to 50 years with a mean age of 21.3 ± SD 10.13 years	Photodynamic therapy (PDT) for a maximum of eight sessions	Patient HDSS and Sweating Intensity Visual Scale of Minor's test	Dropping of HDSS with achievement of Grade 1 in 60% and Grade 2 in 40% of the cases at week 8.	Slight pain and transient discolouration in 95% of the patients	N/A

Campanati et al. 2020 [19]	Prospective, randomized case-control analysis	70	Group 1: 38.01 ± 12.7 Group 2: 37.01 ± 14.6	Group 1 was treated with BTX-A injections at T0, followed by oral administration of oxybutynin chloride at the time of hyperhidrosis relapse. Group 2 was treated from the beginning with oral oxybutynin chloride in monotherapy.	Patient HDSS and DLQI scores Efficacy was evaluated and compared between two groups through the patients' assessment of the tolerability of sweating and interference effect of sweating on daily activities	Group 1: 82% (27/33) of the patients were successfully treated with low dosage of oxybutynin chloride (7.5 mg/die) Group 2: 31% (8/26) of the patients were able to control symptoms with low dosage of oxybutynin chloride, and difference was significant between groups (*p*=0.001).	Group 1: withdrawal of two patients (2/35 = 5.71%) (1 for xerophthalmia, and 1 for vaginal dryness) Group 2: withdrawal of 9 patients (9/35 = 25.71%) (2 for dizziness, 1 for constipation, 5 for xerophthalmia and 1 for vaginal dryness)	Patients were assessed at the baseline, 4 weeks (T4), 24 weeks (T24) and 52 weeks (T52) after treatments

Kim et al. 2017 [20]	Randomized, sham-controlled, single-blind and parallel-designed study	29	Mean age was 30.2 ± 11.7 years	Iontophoresis for 20 min 5 times/week for 2 weeks	Gravimetry and patient QOLQ	Starch–iodine test showed increased clinical improvement, reduced sweating and improved QoL in treatment group	Mild localized hand erythema [1]	Patients were evaluated before treatment and at 2, 3 and 4 weeks

Artzi et al. 2017 [21]	A randomized double-blind placebo-controlled split area study	61	Age ranged from 18 to 52 years with a mean age of 31.6 ± 1.4 years	Topical oxybutynin 10% gel for 4 weeks	Patient HDSS and DLQI scores and satisfaction questionnaires Minor starch–iodine test A blinded visual grading of the photographs taken before and after 30 days of treatment was the primary endpoint.	Overall increased sweat reduction in treated vs. nontreated areas. 79% of the patients correctly identified the treated side. 66% of the patients reported a 1-point reduction in HDSS. 74% of the patients were moderately to highly satisfied following treatment	Transient headaches [2] Erythema and pruritus [11]	Change in perspiration was noted in the treated and control areas as well as in other distant sites

Schlollhammer et al. 2015 [22]	Prospective, randomized, placebo‐controlled trial	62	Age ranged from 18 to 62 years with a mean age of 33.5 years	Oral oxybutynin	Patient HDSS and DLQI	60% of the patients experienced improvement in HDSS treated with oxybutynin, compared with 27% of the patients treated with placebo	Dry mouth in 43% of the patients	N/A

Bell et al. 2014 [23]	Retrospective uncontrolled study	93	Age ranged from 11 to 77 years, with a median age of 28 years	Endoscopic thoracic sympathectomy	Written and telephone QOLQ	97% of the patients reported near-complete symptom resolution of PH	Compensatory sweating in 68% of patients	Patients were given 1-month postsurgery to complete the questionnaire

Wolosker et al. 2014 [24]	Prospective uncontrolled study	570	Age ranged from 4 to 61 years with a mean age of 22 years	Oral oxybutynin	Patient QOLQ and clinical questionnaire	97.2% of the patients experienced improvement in their pH	Dry mouth in 49.6% of patients	Patients were evaluated before treatment and at 6 and 24 weeks

Chia et al. 2012 [25]	Prospective uncontrolled study	25	Age ranged from 13 to 51 years with a mean age of 23.9 years	Iontophoresis	Gravimetry and patient QOLQ	81.8% of patients experienced improvement in their pH	Dry mouth in 100% of patients	Patients were evaluated before treatment and 1 week after starting 4 weeks of treatment

Wolosker et al. 2011 [26]	Prospective uncontrolled study	180	Age ranged from 18 to 56 years with a mean age of 24.4 ± 9.6	Oral oxybutynin	Patient QOLQ and clinical questionnaire	80% of the patients experienced improvement in their pH	Dry mouth in 70.5% of the patients	Patients were evaluated before treatment and at 6 and 12 weeks

Loscertales et al. 2004 [27]	Retrospective uncontrolled study	113	Age ranged from 14 to 50 years of age	Endoscopic thoracic sympathectomy	Patient QOLQ	100% of the patients reported complete symptom resolution of PH	Compensatory sweating in 67% of the patients	Patients were evaluated 1 month and 1 year after surgery

Schnider et al. 1997 [28]	A randomized double-blind placebo-controlled split area study	11	Age ranged from 23 to 54 years with a mean age of 33.2 ± 9.6 years	Botulinum toxin A injection	Patient VAS, satisfaction questionnaire, and digitalized ninhydrin-stained sheets	Patients reported a 38% improvement at 13 weeks of treatment	Mild hand and grip weakness in 27% of patients	Patients were evaluated before, 3, 8 and 13 weeks after treatment

Abbreviations: DLQI, Dermatology Life Quality Index; ETS, endoscopic thoracic sympathectomy; HDSS, Hyperhidrosis Disease Severity Scale; PDT, photodynamic therapy; QOLQ, Quality-of-Life Questionnaire; VAS, Visual Analogue Scale.

## Data Availability

All data analysed during this study are included within the published article and Supporting files. Further enquiries can be directed to the corresponding author.

## Supporting Information

Table 2.0 illustrates the met hodological quality of the studies using the Cochrane Collaboration's tool can be found in the Supporting section.
