# Supplementary material for: Efficacy and Safety of Treatments for Primary Palmar Hyperhidrosis: A Systematic Review Assessing Patient-Centric Outcomes
Source: Dermatol Res Pract. 2025 Aug 20;2025:8867838. doi: 10.1155/drp/8867838 (PMC12390518; doi:10.1155/drp/8867838)
Supplement: Supporting Information — Additional supporting information can be found online in the Supporting Information section. [file 8867838.f1.docx]

| **Author** | **Random sequence generation** | **Allocation Concealment** | **Blinding of participants and personnel’s** | **Blinding of outcome assessment** | **Incomplete outcome data** | **Selective Reporting** | **Other bias** | **Total out of 14** | **Percentage conversion** | **Risk of bias status** |
| --- | --- | --- | --- | --- | --- | --- | --- | --- | --- | --- |
| Markantoni et al. 2023 (15) | 1 | 1 | 2 | 2 | 2 | 2 | 0 | 10 out of 14 | 71.43% | Moderate |
| Saki et al. 2023 (16) | 1 | 1 | 2 | 2 | 2 | 2 | 0 | 10 out of 14 | 71.43% | Moderate |
| Zhang et al. 2022 (17) | 1 | 1 | 2 | 2 | 1 | 1 | 0 | 8 out of 14 | 57.14% | High |
| Shabaik et al. 2021 (18) | 1 | 0 | 1 | 1 | 1 | 2 | 0 | 6 out of 14 | 42.68% | High |
| Campanati et al. 2020 (19) | 1 | 1 | 1 | 2 | 2 | 2 | 0 | 9 out of 14 | 64.29% | Moderate |
| Kim et al. 2017 (20) | 1 | 1 | 1 | 2 | 2 | 2 | 0 | 9 out of 14 | 64.29% | Moderate |
| Artzi et al. 2017 (21) | 1 | 1 | 2 | 2 | 2 | 2 | 0 | 10 out of 14 | 71.43% | Moderate |
| Schlollhammer et al. 2015 (22) | 1 | 1 | 2 | 2 | 2 | 2 | 0 | 10 out of 14 | 71.43% | Moderate |
| Bell et al. 2014 (23) | 0 | 0 | 0 | 1 | 1 | 2 | 1 | 5 out of 14 | 35.71% | High |
| Wolosker et al. 2014 (24) | 0 | 0 | 0 | 1 | 1 | 2 | 1 | 5 out of 14 | 35.71% | High |
| Chia et al. 2012 (25) | 1 | 1 | 2 | 2 | 2 | 2 | 0 | 10 out of 14 | 71.43% | Moderate |
| Wolosker et al. 2011 (26) | 0 | 0 | 0 | 1 | 1 | 2 | 1 | 5 out of 14 | 35.71% | High |
| Loscertales et al. 2004 (27) | 0 | 0 | 0 | 1 | 1 | 2 | 1 | 5 out of 14 | 35.71% | High |
| Schnider et al. 1997 (28) | 1 | 1 | 2 | 2 | 2 | 2 | 0 | 10 out of 14 | 71.43% | Moderate |

**Table 2.0:** Methodological quality and risk of bias using the Cochrane Collaboration’s tool (12)
